# Degradation characteristics of biodegradable film and its effects on soil nutrients in tillage layer, growth and development of taro and yield formation

**DOI:** 10.1186/s13568-022-01420-y

**Published:** 2022-06-22

**Authors:** An Wang, Qingtao Chang, Chunsheng Chen, Xiaoquan Zhong, Kexiang Yuan, Meihua Yang, Wei Wu

**Affiliations:** 1 Special Grain Classics Laboratory, Taizhou Institute of Agricultural Science, Jiangsu Academy of Agricultural Sciences, 56 Autumn Snow Lake Avenue, Taizhou, 225300 China; 2 Department of Vegetable, Xinghua Modern Agriculture Development Service Center, Taizhou, 225700 China; 3 Xinghua Meihua Vegetable Planting Cooperative, Taizhou, 225700 China

**Keywords:** Biodegradable film, Taro longxiang, Soil fertility, Yield, Quality

## Abstract

This study investigated the degradation characteristics of different biodegradable film and its effects on soil nutrients in tillage layer, growth and development of taro and yield formation**.** Field experiment with biodegradable films, including poly-(butylene adipate-co-butylene terephthalate) PBAT, (poly-carbon dioxide) PCO2, (poly propylene carbonate) PPC, as well as common mulch film (CK1) and uncovered mulch film (CK2) were conducted on Longxiang taro in 2020 and 2021 respectively. The degradation rate of the three biodegradable films was PBAT > PPC > PCO2. Compared with CK1, the alkali-hydrolyzed N of PBAT at the growth stage and fruiting stage significantly increased in 2020 and 2021, respectively (both, *P* < *0.05*). The average content of available P of PPC at seedling stage was higher than that in PCO2, and CK1 was significantly decreased compared with that in CK2 (all, *P* < *0.05*). The content of soil available K and organic matter in different growth stages of taro in all film mulching treatments were decreased in comparison to CK2. Moreover, compared with CK2, PCO2 biodegradable film significantly increased plant height at seedling and growth stage, stem diameter at growth stage, and leaf area index at fruiting stage (all, *P* < *0.05*). Similarly, the yield of mother and filial bulbs of PPC, PCO2 and PBAT were significantly higher than those of CK2 in 2020 and 2021, respectively (all, *P* < *0.05*). However, no significant differences were found in starch, polysaccharide and protein contents among different treatments. The three biodegradable films, especially PCO2, can significantly affect soil nutrient content, promote plant growth and improve taro yield.

**Supplementary Information:**

The online version contains supplementary material available at 10.1186/s13568-022-01420-y.

## Introduction

Taro (*Colocasia esculenta (L.) Schott*), the most important crop of *Araceae* family, has important edible, medicinal, nutritional and economic values (Yin et al. 2021). Native to swampy tropical regions such as China, India and Malaysia, taro is a staple food for nearly 1% of the world's population (Singla et al. 2020). In China, taro is also one of the important local characteristic crops in Taizhou, Nantong, Changzhou and other areas along the Yangtze River in Jiangsu Province. And the annual planting area about 13,300 hm^2^, and the varieties planted are mostly *multiseed* taro (Wang et al. 2021a), such as *Jingjiang Xiangsha Arum*, *Haimen Wannian Arum*, and *Changzhou Hongxiang Arum*. Usually, taro is a warm-loving crop, and low temperature conditions are not conducive to taro germination and growth (Bellinger et al. 2020). Film mulching can improve soil temperature by inhibiting heat exchange between soil and atmosphere, and is an important technical measure to improve crop growth potential and yield formation (Chen et al. 2021; Lee et al. 2021). However, traditional mulching film mainly uses polyethylene as raw material, and due to its stable molecular structure and non-perishable decomposition characteristics, perennial use will lead to the accumulation of soil residual film, posing a serious threat to the sustainable development of agriculture.

During the past decades, biodegradable film has becoming one of the effective ways to solve the problem of white pollution in agriculture. The advantages of biodegradable film has been reported as follows: improved tensile strength, flexibility, and controllability of rupture and degradation; pollution free; enhanced ability to increase the soil temperature and preserve the soil moisture and satisfy the demand of crop production with mulching (Yan et al. 2016). Till now, an increasing number of researches on biodegradable film are carried out in different crops, such as corn (Chen et al. 2021; Wang et al. 2021b), wheat(Qi et al. 2020), rice(Yao et al. 2017), and cutton (Wang et al. 2018). But till now, there are few reports about biodegradable films on taro.

Polybutylene adipate terephthalate (PBAT) is a biodegradable aliphatic polyester, which has been widely used in many fields due to its good ductility and elongation at break, good heat resistance and processing performance (Ferreira et al. 2019). In particular, PBAT is a potentially effective choice of agricultural mulching film degradation material due to its characteristics of fast degradation rate, high degradation rate and less residue (Serrano-Ruíz et al. 2018; Zhang et al. 2019). Poly propylene carbonate (PPC) is a completely biodegradable, tough aliphatic carbonate polyol with excellent barrier properties. Moreover, PPC is an amorphous polymer, which is considered a good candidate for blending with PCL to expand the applications of both (Cheng et al. 2022). As a new material developed by Nantong Longda Biological New Material Technology Co., LTD, the main decomposition product of PCO2 (poly-carbon dioxide) is CO_2_. However, the application of PCO2 in agricultural production has not been widely reported till now. In the present study, we investigated the degradation characteristics of different biodegradable film and its effects on soil nutrients in tillage layer, growth and development of taro and yield formation, which will provide theoretical and practical basis for the application of biodegradable film in sustainable ecological cultivation of taro.

## Materials and methods

### Study site and experimental design

The study was carried out in Longxiang taro demonstration base during 2020 and 2021 at the Xinghua qianduo town Chaigou village, JiangSu, China (119°45′17 "N、32°59′29 "E). The experimental site belongs to the north subtropical humid monsoon climate zone, with an average altitude of 1.84 m, and the average annual temperature, precipitation and rain days are 15.0 ℃, 1032.3 mm and 109 d (Additional file 1: Figure S1), respectively. The soil in this area was sticky, and the contents of organic matter, alkali-hydrolyzable nitrogen, available phosphorus and available potassium in the plough layer (0–20 cm) were 15.87 g·kg^−1^, 37.64 mg·kg^−1^, 21.45 mg·kg^−1^ and 67.67 mg·kg^−1^ in 2020, as well as 16.73 g·kg^−1^, 32.48 mg·kg^−1^, 20.28 mg·kg^−1^ and 62.41 mg·kg^−1^ in 2021, respectively.

Xinghua longxiang arum, provided by Xinghua Meihua Vegetable Planting Cooperative, was used as the test material. Three biodegradable films were selected in the experimental groups as follows: PPC biodegradable film (Zhongke Jinlong Co., LTD.), PCO2 biodegradable film (Nantong Longda Biological New Material Technology Co., LTD), and PBAT biodegradable film (Shanghai Hongrui Biotechnology Co., LTD.); moreover, mulch film (CK1) and open ground (CK2) as the control. The specifications of the film were 120 cm wide and 0.008 mm thick. All treatments were laid out as randomized block designs with three replicates, where the length was 6 m, the width was 3.6 m, and the area was 21.6 m^2^.

In this study, furrow cave-planting method was adopted. First, deep tillage will be carried out for frozen-tillage before winter; before rotation tillage, K_2_SO_4_ compound fertilizer (n-p2o5-k2o,15-15-15) and commercial organic fertilizer (organic matter ≥ 29%, total nitrogen 3%, total phosphorus 1.2%, total potassium 2.2%) will be applied at the rate of 600 kg and 15 t/hm^2^, respectively. Second, the seed taro was sown in the direction of the furrow, with a spacing of 45 cm and a row spacing of 70 cm. Third, after sowing, the seed taro was covered with film, and the film edge should be compacted with soil. Then, film was broken and taro seedlings were released in time after emergence, and K_2_SO_4_ compound fertilizer was applied between two taro plants at the point of taro forming stage with the dosage of 450 kg hm^−2^. Other field management was the same as local field production. In 2020, the planting date was April 7 and the harvest date was November 2; and the planting date for 2021 was April 2 and the harvest was October 30.

### Sampling and measurements

#### Degradation characteristics of biodegradable film

There are five stages of plastic film degradation as follows: (a) Induction stage: from plastic film mulching to the stage when there are many natural cracks or holes (more than 3 per meter) < 2 cm on the furrow surface; (b) Cracking stage: the period of 2 ~ 20 cm natural cracks or holes (diameter) in the furrow surface mulching film; (c) Large crack stage: the screen surface mulching film appears more than 20 cm natural crack or hole (diameter) period; (d) Fragmentation stage: the period when the mulching film on the bed surface is fragmented and the maximum residual area of mulching film is ≤ 16 cm^2^; (e) Film-free stage: the strip surface can not see the film residue.

### Determination of soil nutrients

Soil samples (0 ~ 20 cm) were collected at seedling stage, growing stage and setting stage, respectively. Moreover, the contents of alkali-hydrolyzed nitrogen (alkali-hydrolyzed diffusion method), available phosphorus (molybdenum-antimony resistance colorimetric method), available potassium (flame photometry method) and organic matter (hydration heat method) (Lu 2000) in the soil were determined after the soil was dried naturally.

### Plant morphological index

Five representative plants with uniform growth were randomly selected from each plot at the seedling stage, initiation stage and setting stage, and their plant height, stem diameter and leaf area index (LAI) were measured. The LAI calculation formula was as follows:

$${\text{LAI = 0}}{.75}\rho \frac{{\sum\limits_{{\text{j = 1}}}^{{\text{m}}} {\sum\limits_{{\text{i = 1}}}^{{\text{n}}} {\left( {L_{ij} \times {\text{B}}_{ij} } \right)} } }}{m}$$

M is the number of measured plants, n is the total number of leaves of j plant, ρ is planting density, Lij is the length of corresponding leaves of each plant, and Bij is the width of corresponding leaves of each plant.

### Production

In the mature stage, the taro of each plot was dig out, and the soil and root system on the surface of taro were removed. Next, the weight of the mother taro and the seed taro were measured after drying, respectively.

### Quality determination

After drying and peeling, the starch, polysaccharide and protein in the center of taro bulb were determined using iodine colorimetric method, acid-hydrolyzed phenol–sulfuric acid colorimetric method, and Coomassie bright blue colorimetric method.

### Statistical analysis

All data in this study was analyzed using GraphPad Prism (verson 8.0.0, GraphPad Software, San Diego, California USA, www.graphpad.com). Differences in different groups were analyzed by one-way analysis of variance (ANOVA), followed by Dunnett multiple comparisons. A *P* value < 0.05 was considered statistically significant.

## Results

### Degradation characteristics of biodegradable films

As shown in Table 1, the degradation rates of different biodegradable films were different, and the overall degradation rate of each treatment was PPC > PBAT > PCO2. All the three biodegradable films could degrade naturally at the later growth stage of taro, among which, PPC and PBAT entered the film-free stage at 130d and 118.5d after coating. PCO2 entered the film-free stage at 153.5 days, which was more than 20 days longer than PPC and PBAT; meanwhile, compared with PPC and PBAT, PCO2 from induction stage to film-free stage was prolonged by 9 and 26 days in 2020, as well as 10 and 29 days in 2021, respectively.

**Table 1:** Degradation characteristics of different biodegradable films

Year	Treatment	Induction stage	Cracking stage	Large crack stage	Fragmentation stage	Film-free stage
2020	PPC	43	62	77	89	128
	PCO2	55	74	89	104	149
	PBAT	46	57	62	76	114
2021	PPC	46	71	88	101	135
	PCO2	59	89	104	121	158
	PBAT	53	63	71	87	123

In addition, compared with 2020, the degradation cycle of PPC, PCO2 and PBAT biodegradable films in 2021 was 7, 9 and 9d longer, respectively. And we speculated that the precipitation in June and July 2020 increased by 61.78% and 22.52%, respectively, compared with the same period in 2021, leading to the early cracking stage and big cracking stage of biodegradable films.

### Effects of biodegradable film mulching on soil nutrients

#### Soil alkali-hydrolyzable nitrogen (N)

**Fig. 1:**
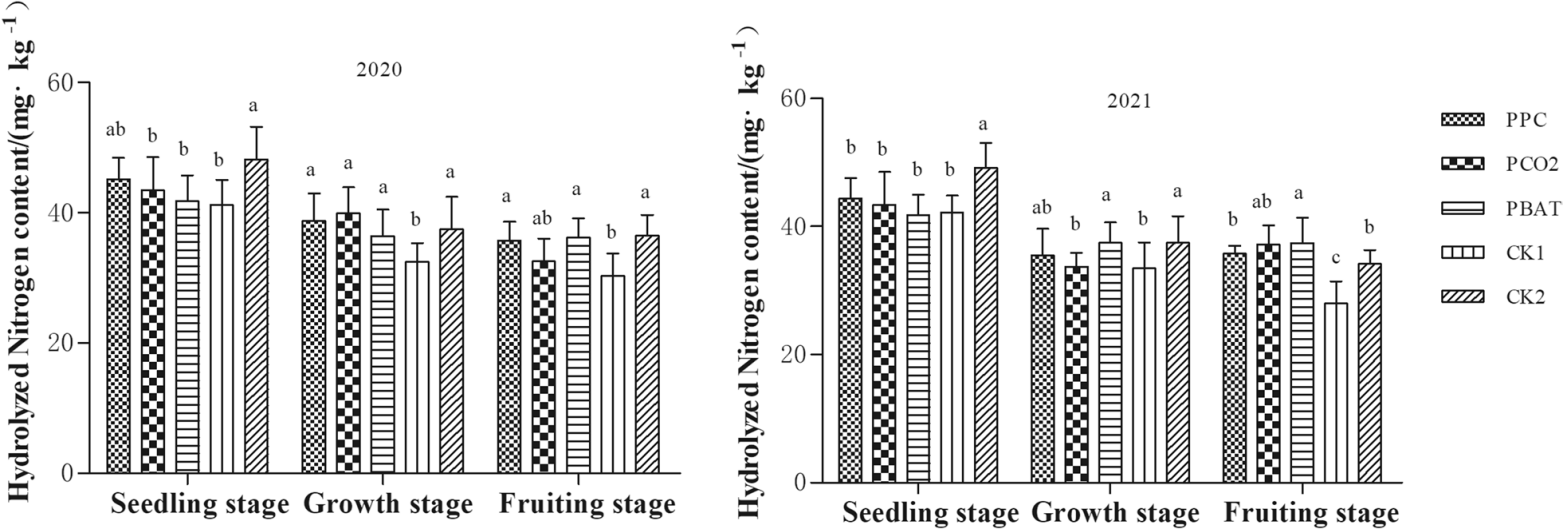
Hydrolyzed Nitrogen content in soil of different mulching treatment. Different lowercase letters indicate significant difference between treatments (P < 0. 05)

In 2020, soil N content of different treatments showed a gradually decreasing trend (except PBAT) with the advance of the growth period of taro (Fig. 1). At seedling stage, the N content in CK2 was the highest, which was 4.79, 6.39 and 7.03 mg·kg-1 higher than that in PCO2, PBAT and CK1, respectively. However, there was no significant difference in soil N content among different film mulching treatments. At the growth stage, soil N content in CK1 (32.4 mg·kg^−1^) was the lowest, which was significantly lower than that in other treatments (all, P < 0.05). Similarly, soil N content was the lowest in CK1, with an average of 30.28 mg·kg^−1^ at taro setting stage, which was 17.89, 19.39 and 20.48% lower than that in PPC, PBAT and CK2 (all, P < 0.05), respectively. However, there was no significant difference in the N content in other three biodegradable membrane soils.

In 2021, the difference trend of soil N between different treatments was basically the same as that in 2020, except that the difference of PCO2 between other treatments was different from that in 2020.

#### Soil available phosphorus (P)

**Fig. 2:**
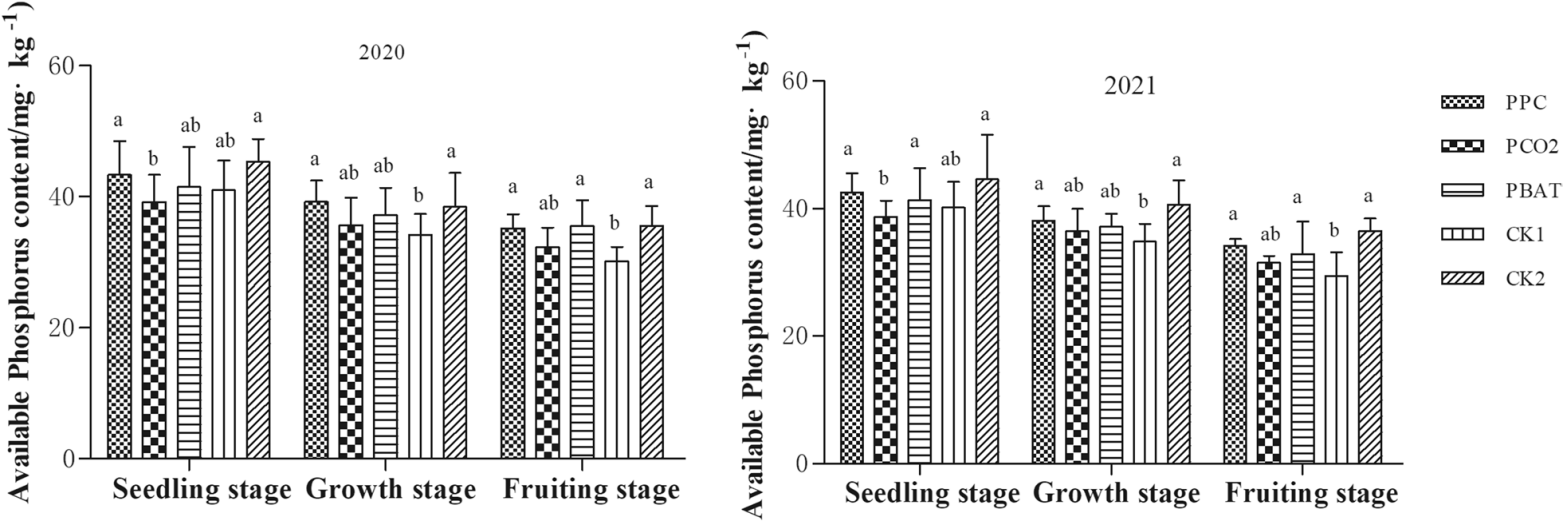
Available Phosphorus content in soil of different mulching treatment. Different lowercase letters indicate significant difference between treatments (P < 0. 05)

As illustrated in Fig. 2, with the advance of taro growth, available P content in soil of all treatments decreased gradually, and reached the lowest in taro setting stage. In 2020, the soil available P of PCO2 was the lowest at seedling stage, with an average content of 39.15 mg/kg^−1^, which was 4.18 and 6.17 mg·kg^−1^ lower than that of PPC and CK2, respectively. In 2021, the soil available P of PCO2 was significantly reduced in comparison to that in PPC, PBAT and CK2 at seedling stage (all, P < 0.05). Moreover, there was no significant difference in the content of available P in the three kinds of biodegradable film soil at the growth stage and the taro setting stage, respectively. Compared with CK1, soil available P content in PPC at growth stage, as well as in PPC and PBAT at the setting stage were significantly decreased (all, P < 0.05).

#### Soil available Potassium (K)

**Fig. 3:**
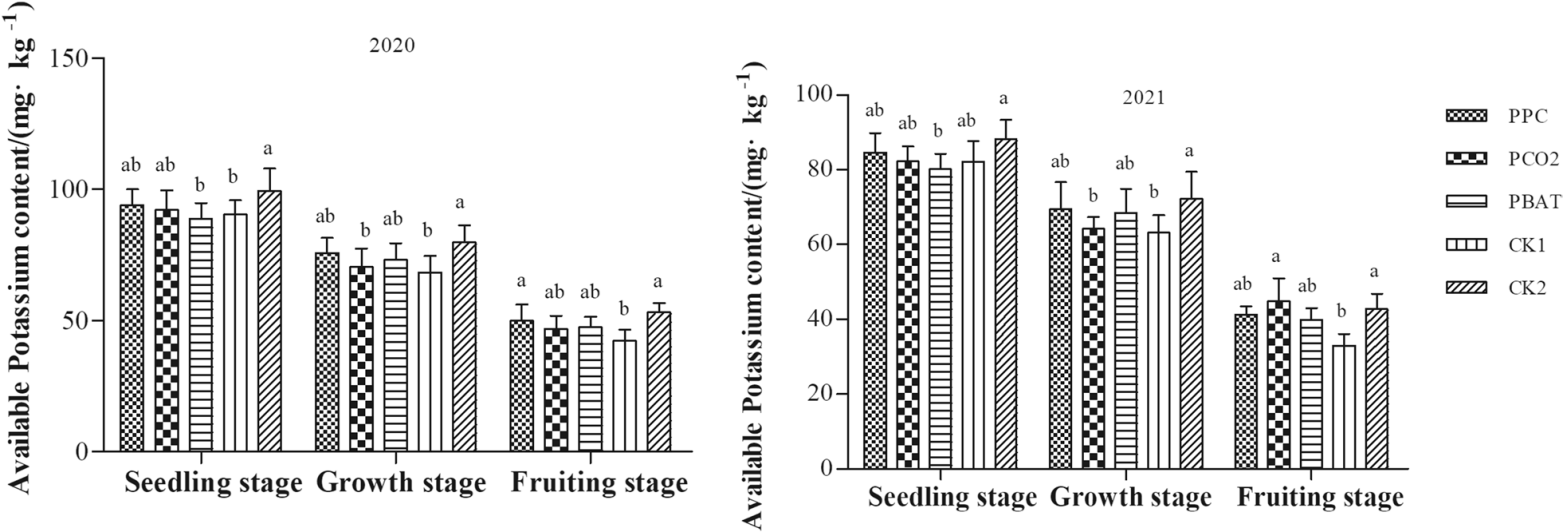
Available Potassium content in soil of different mulching treatment. Different lowercase letters indicate significant difference between treatments (P < 0. 05)

In line with soil N and available P, the available K content in soil of all treatments decreased gradually with the advance of taro growth, and reached the lowest at taro setting stage (Fig. 3), indicating that the soil available K was consumed by the underground bulb of taro. In 2020 or 2021, there was no significant difference in soil available K content between the three biodegradable films at seedling stage, growth stage and taro setting stage. Furthermore, the available K content of PBAT at seedling stage, as well as PCO2 and CK1 at growth stage were observably lower than those in CK2 (all, P < 0.05). At taro setting stage, when compared with CK1, the available K content of PPC in 2020, and PCO2 and CK2 in 2021 were significantly increased (all, P < 0.05).

### Soil organic matter content

**Fig. 4:**
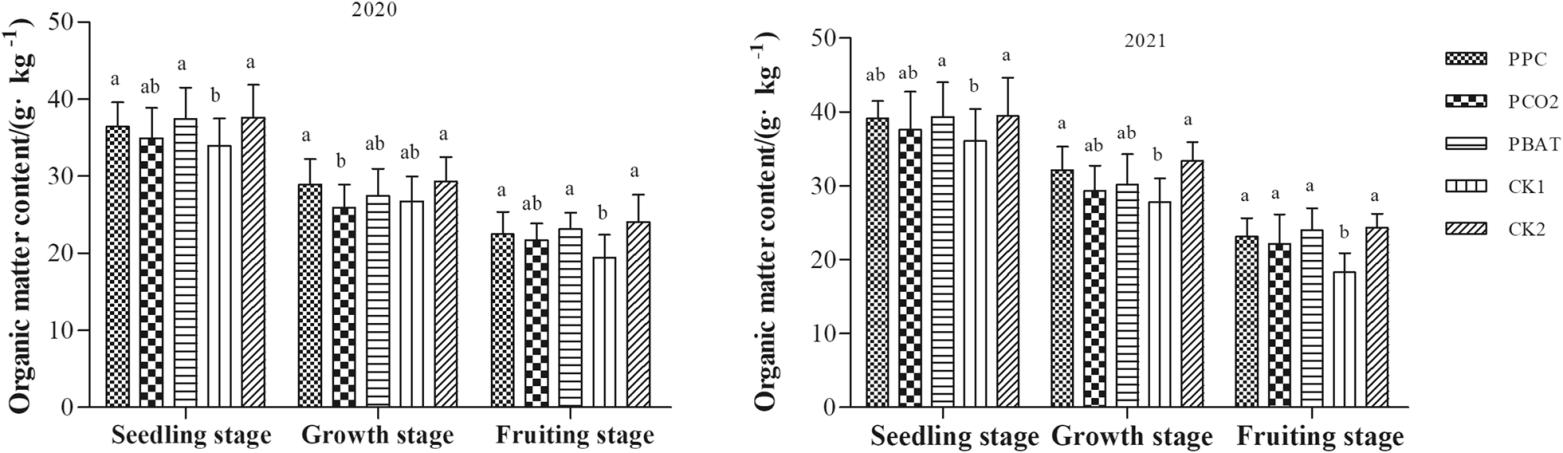
Organic matter content in soil of different mulching treatment. Different lowercase letters indicate significant difference between treatments (P < 0. 05)

According to Fig. 4, the content of soil organic matter in all treatments decreased gradually with the development of growth period. And no significant difference was found in soil organic matter content between the three biodegradable films at seedling stage in 2 years (all, P > 0.05). Moreover, compared with CK1, soil organic matter content in PBAT and CK2 increased by 10.21 and 10.74% (2020), and 9.18 and 9.51% (2021), respectively (all, P < 0.05). At growth stage, PCO2 decreased by 3 and 2.6 g·kg^−1^ compared with PPC and CK2 in 2020; meanwhile, CK1 decreased by 4.31 and 5.6 g·kg^−1^ compared with PPC and CK2 in 2021, respectively (all, P < 0.05). At the fruiting stage, the soil organic matter content of CK1 was 19.49 g·kg^−1^ and 18.32 g·kg^−1^, which was significantly lower than that of other mulching treatments (all, P < 0.05).

### Effects of biodegradable films on morphological characteristics of taro

The results showed the effects of film mulching on plant height, stem diameter and leaf area index were significant (Table 2). Briefly, the order of plant height at seedling stage and growth stage was CK1 > PCO2 > PBAT > PPC > CK2, indicating that film mulching treatment had the most significant effect on promoting the growth of taro. At seedling stage, the plant height of PCO2 increased by 8.78 (2020) and 10.41% (2021) compared with PPC, respectively (both, P < 0.05). The plant height of CK1 was significantly higher than that of other treatments at the stage of fruiting. Moreover, when compared with CK2, the plant height of PPC and PCO2 was significantly upregulated at the stages of growth and fruiting in both years, respectively (all, P < 0.05).

**Table 2:** Main morphological indexes of taro between different plastic mulching

Year	Treatment	Plant height/ (cm)			Thick stems/(cm)			Leaf area index		
		Seeding stage	Growth stage	Fruiting stage	Seeding stage	Growth stage	Fruiting stage	Seeding stage	Growth stage	Fruiting stage
2020	PPC	35.42 c	72.26 b	116.87 ab	3.34 b	7.52 a	9.72 b	0.45 a	1.71 ab	3.28 ab
	PCO2	37.95 b	78.15 ab	118.64 a	3.49 ab	7.76 a	10.15 ab	0.48 a	1.62 b	3.38 a
	PBAT	36.48 b	74.16 b	114.25 ab	3.45 b	7.02 ab	9.68 b	0.41 ab	1.66 ab	3.24 ab
	CK1	39.12 a	83.26 a	125.32 a	4.04 a	8.29 a	11.34 a	0.52 a	1.88 a	3.46 a
	CK2	33.12 c	70.12 b	105.32 b	3.25 b	6.54 b	8.42 c	0.41 b	1.56 b	3.08 b
2021	PPC	35.46 b	74.46 ab	114.37 a	3.59 bc	7.73 b	9.12 b	0.46 a	1.78 b	2.98 ab
	PCO2	39.15 a	80.85 a	115.64 a	3.79 ab	7.95 ab	9.89 ab	0.42 b	1.88 ab	3.11 a
	PBAT	37.64 ab	76.66 ab	112.25 a	3.7 b	7.33 bc	9.38 b	0.44 ab	1.86 ab	3.04 a
	CK1	42.12 a	85.66 a	116.32 a	4.29 a	8.52 a	10.77 a	0.49 a	2.09 a	3.31 a
	CK2	31.15 c	71.62 b	101.32 b	3.49 c	6.72 c	8.24 c	0.38 b	1.68 b	2.84b

Different lower-case letters denote significant differences among treatments

Additionally, there was no significant difference in stem diameter between the three biodegradable files and CK2 at different growth stages (all, P > 0.05). When compared with CK2, the leaf area index at seedling stage of PPC increased by 9.76 (2020) and 21.05% (2021), respectively (both, P < 0.05). There was no significant difference between the leaf area index of each biodegradable film at the stages of growth and fruiting. No significant difference between the leaf area index of each biodegradable film and CK2 was found at growth stage, and the leaf area index of PCO2 at fruiting stage was significantly higher than that of CK2, with an increase of 9.74 (2020) and 9.15% (2021), respectively (both, P < 0.05).

### Effect of biodegradable films on yield of taro

**Fig. 5:**
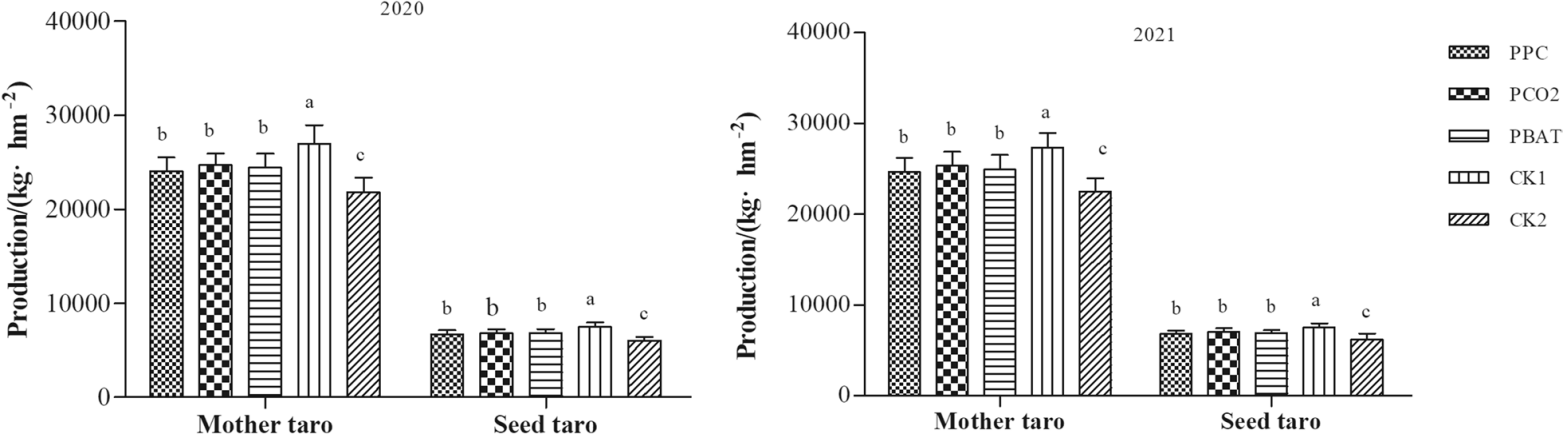
The taro longxiang production of different biodegradable films. Different lowercase letters indicate significant difference between treatments (P < 0. 05)

The yield of mother taro and seed taro of different treatments for two years was measured, respectively. The results demonstrated that the yield of mother taro and seed taro was consistent in two years, with the yield order of CK1 > PCO2 > PBAT > PPC > CK2 (Fig. 5); however, the difference in the yield of mother taro and seed taro among the three biodegradable films was not significant. Among them, the yields of mother taro and seed taro of CK1 were significantly higher than those of the three biodegradable films. Furthermore, when compared with CK2, the average yield of mother taro and seed taro in PPC, PCO2 and PBAT increased by 2200.35, 2868.45 and 2516.1 kg·hm^−2^, as well as 674.37, 822.45 and 771.99 kg·hm^−2^, respectively (all, P < 0.05).

### Effect of biodegradable films on quality index of taro

To further evaluate the quality of mother taro, the contents of starch, polysaccharide and protein in taro were measured in this study. As shown in Table 3, the films mulching had no significant effect on the contents of starch, polysaccharide and protein in taro. During the two years, the highest starch content was found in CK1 with 131.76 mg·g^−1^ (2020) and 135.48 mg·g^−1^ (2021), and the lowest starch content was found in CK2 with 128.39 mg·g^−1^ (2020) and PBAT with 130.54 mg·g^−1^ (2021); however, the difference was not significant (all, *P* < 0.05).

**Table 3:** Analysis of quality indexes of Taro with different treatments

Year	Treatment	Starch/(mg·g^−1^)	Polysaccharide/(mg·g^−1^)	Protein/(mg·g^−1^)
2020	PPC	125.46 a	28.71 a	33.64 a
	PCO2	130.24 a	26.94 a	35.02 a
	PBAT	128.45 a	27.54 a	33.98 a
	CK1	131.76 a	28.18 a	34.15 a
	CK2	128.39 a	27.79 a	34.19 a
2021	PPC	134.25 a	30.51 a	32.15 a
	PCO2	133.49 a	29.46 a	34.02 a
	PBAT	130.54 a	29.15 a	33.78 a
	CK1	135.48 a	31.08 a	32.81 a
	CK2	134.28 a	30.88 a	33.46 a

Different lower-case letters denote significant differences among treatments

## Discussion

### Field degradation characteristics and influencing factors of biodegradable film

Generally, the degradation rate and degradation cycle of biodegradable films in the field are mainly affected by a variety of factors such as film material composition (such as PPC, PBAT, PLA, etc.), film thickness, and ecological environment (Diao et al. 2022; La Mantia et al. 2020; Sumayya et al. 2021; Zhao et al. 2021). Therefore, in crop production, it is an effective way to promote crop growth and reduce field residual film pollution to screen out biodegradable films with appropriate degradation rate and cycle by combining the factors of plant growth rule, local cultivation mode and ecological climate characteristics (Hu et al. 2020; Khan et al. 2020; Rosseto et al. 2019). Our previous study found that PPC biodegradable film had earlier induction period, faster degradation rate and higher degradation degree than PCO2, and its potential effect on increasing production decreased accordingly(Hu et al. 2020; Wang et al. 2021a). In fact, different cultivation methods, fertilization patterns and climatic factors have a significant impact on the growth rate of taro. Hence, we will pay more attention to the correlation analysis between the degradation rate of biomulching films and other factors that affect the growth rate of taro in the future study.

In the present study, the findings revealed that different biodegradable films had different degrees of field degradation, which may be caused by different formulations of biodegradable films leading to different effects of soil microorganisms on them (Eksiler et al. 2017; Xue et al. 2021). Additionally, the degradation of three biodegradable films began to be induced at 43 ~ 59d after mulching, and large cracks appeared at 62 ~ 104d after mulching. When the integrity of mulching film was destroyed, the degradation rate of the film was accelerated, and the degradation cycle was shorter, and basically no agricultural film residue was left in the field at the maturity stage. Compared with PPC and PBAT biodegradable films, PCO2 had a longer induction period, longer degradation cycle, and the lowest degradation rate, which is similar to our previous report (Wang et al. 2021a). Moreover, due to the difference of precipitation between 2020 and 2021, PPC, PCO2 and PBAT biodegradable films performed different degradation time, and their stability and degradation rate have different degrees of change. For example, the induction period of the three biodegradable films in 2020 was 3-7d earlier than that in 2021, and the film-free period was 11-17d earlier than that in 2021. Actually, the degradation rate of biodegradable film is usually proportional to rainfall, so the degradation rate will be different under different climatic conditions (Feng et al. 2019).

### Effects of biodegradable film mulching on soil nutrient contents

Soil nutrient is one of the main indexes to measure soil fertility, and its content directly affects soil fertility supply and crop growth (Ghosh et al. 2022). Organic matter, N, P and K content are the main sources of soil nutrients, which are the basis of nutrient absorption and utilization for crop maintenance (Babu et al. 2020). Accumulating evidence has shown that the films mulching can effectively improve soil aggregate structure, bulk density, porosity and other physical structures (Tang et al. 2022). Furthermore, it can improve the activity of soil enzymes, accelerate the decomposition of nutrients by microorganisms, accelerate the mineralization process of soil organic matter, promote the efficient absorption and utilization of soil nutrients by crops, and ultimately affect soil nutrient content (Tang et al. 2022; Yang et al. 2020). The data of this study showed that the contents of alkali-hydrolyzable N, available P, available K and organic matter in soil of film mulching treatments had a decreasing trend in different growth stages of taro compared with CK2, indicating that the absorption and utilization of soil nutrients in open-field cultivation was lower than that of film mulching.

In addition, PPC, PCO2 and PBAT biodegradable films showed little difference in soil alkal-hydrolyzable N, available P, available K and organic matter nutrient contents, but the nutrient contents were higher than CK1 at the same time, indicating that the promoting effect of biodegradable film on soil nutrient release was lower than that of ordinary plastic film, which was different from the report of Wang et al.(Wang et al. 2019). We speculate that these inconsistent results may be related to the degradation film materials, climatic characteristics, soil types, crop types and other factors.

### Effects of biodegradable film mulching on crop growth and yield

The change of soil microenvironment caused by plastic film mulching performs significant effect on crop growth. An increasing number of researches reveal that both biodegradable film and ordinary plastic film mulching could improve soil water temperature environment during crop growth period, and significantly improve crop agronomic traits such as stem diameter, plant height and leaf area index(Li et al. 2021; Ren et al. 2022; Yang and Gao 2022). In accordance with previous reports, our data suggested when compared with CK2 treatment, the film mulching treatments could significantly promote the growth of taro; meanwhile, the effect of ordinary plastic film mulching on the morphological characteristics of taro plants was significantly higher than that of three biodegradable films. Among them, the difference of plant height in each growth stage reached a significant level, which may be due to the degradation of biodegradable film, the function of temperature increase and moisture retention of plastic film was decreased compared with the ordinary plastic film, and it was difficult to meet the physiological requirements of soil water and temperature of the roots of taro (Feng et al. 2021). Moreover, the soil nutrient content of each biodegradable film was higher than that of ordinary plastic mulch, which indicates that the plants covered by ordinary plastic mulch have higher absorption efficiency of soil nutrient, which may be one of the reasons. In this study, PCO2 had the most significant effect on plant height, stem diameter and leaf area index, which may be related to its slow degradation process.

Till now, there are controversies about the effects of biodegradable film and ordinary mulch film on crop yield. For example, Yang et al. has reported biodegradable film mulching had higher yield increase effect than ordinary film mulching (Yang et al. 2019). According to the study of Feng et al. (Feng et al. 2021), PBAT biodegradable film has no significant difference with ordinary mulching film on maize yield. The data of this study revealed the yield increasing effect of biodegradable film was significantly lower than that of ordinary mulch film, which was similar to the previous study of Gao et al. (Gao et al. 2022). All these findings showed that the application of biodegradable film on taro still needed further improvement, and the main improvement direction was to prolong the effective covering time and maintain stable mechanical strength, especially to ensure that it would not break in the climate environment with high precipitation intensity.

In conclusion, this study comprehensively studied the degradation characteristics of biodegradable film and its effects on the growth, development and yield of taro, and the findings were as follows: (1) PPC, PBAT and PCO2 biodegradable films could be completely degraded during the whole growth period of taro; moreover, PCO2 had the slowest degradation rate, followed by PBAT, and PPC. (2) Compared with CK2, films mulching accelerated the absorption of alkali-hydrolyzed N, available P and available K, and promoted plant height, stem diameter and leaf area index at each growth stage of taro, especially the ordinary mulching film. (3) There was no significant difference in the yield of mother taro and seed taro in three biodegradable films treatments, but they were significantly higher than CK2 treatment and lower than CK1 treatment. (4) No significant differences were found in starch, polysaccharide and protein contents among different treatments. Taken together, the effect of PCO2 biodegradable film mulching was better than that of open field treatment; however, the promotion and application of PCO2 biodegradable film need to be further improved due to the significant difference in yield between PCO2 biodegradable film and ordinary plastic film.

## Supplementary Information

**Additional file 1: Figure S1** The monthly mean temperature (line) and monthly accumulated precipitation (bar) during the growing season of taro longxiang in 2020 and 2021.

## Data Availability

Corresponding authors may provide data and materials where reasonable.

## Abbreviations

PBAT: Polybutylene adipate terephthalate
PPC: Poly propylene carbonate
PCO2: Poly-carbon dioxide
CK1: Common mulch film
CK2: Uncovered mulch film
