# Supplementary figures and images for: Degradation characteristics of biodegradable film and its effects on soil nutrients in tillage layer, growth and development of taro and yield formation
Source: AMB Express. 2022 Jun 22;12:81. doi: 10.1186/s13568-022-01420-y (PMC9218028; doi:10.1186/s13568-022-01420-y)

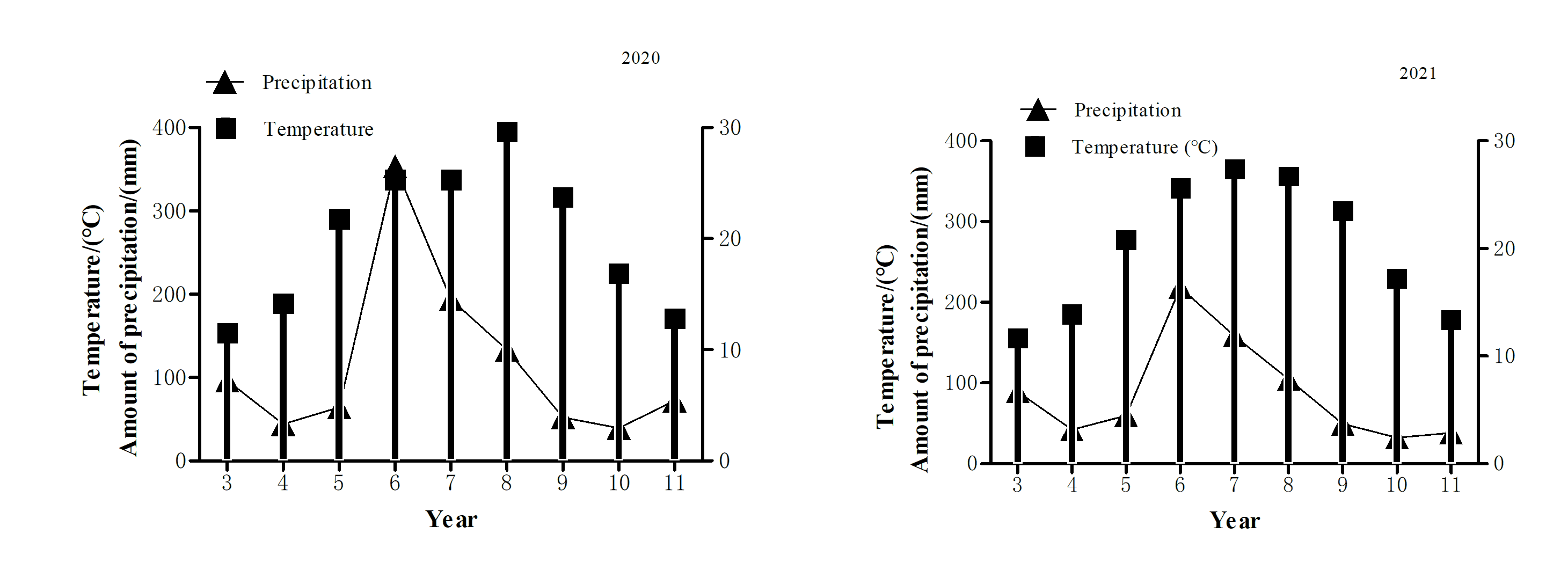

Supplement: Supplementary file 1 — Additional file 1: Figure S1 The monthly mean temperature (line) and monthly accumulated precipitation (bar) during the growing season of taro longxiang in 2020 and 2021. [file 13568_2022_1420_MOESM1_ESM.tif]
